# Proton Pump Inhibitor Use and Efficacy of Nivolumab and Ipilimumab in Advanced Melanoma

**DOI:** 10.3390/cancers14092300

**Published:** 2022-05-05

**Authors:** Krisztian Homicsko, Reinhard Dummer, Christoph Hoeller, Jedd D. Wolchok, F. Stephen Hodi, James Larkin, Paolo A. Ascierto, Victoria Atkinson, Caroline Robert, Michael A. Postow, Sandra Re, David Paulucci, Darin Dobler, Olivier Michielin

**Affiliations:** 1 Department of Oncology, Centre Hospitalier Universitaire Vaudois (CHUV), 1011 Lausanne, Switzerland; 2 Department of Dermatology, Universitäts Spital, 8091 Zurich, Switzerland; reinhard.dummer@usz.ch; 3 Department of Dermatology, Medical University of Vienna, 1090 Vienna, Austria; christoph.hoeller@meduniwien.ac.at; 4 Human Oncology and Pathogenesis Program, Memorial Sloan Kettering Cancer Center, New York, NY 10065, USA; wolchokj@mskcc.org; 5 Department of Medicine, Weill Cornell Medical College, New York, NY 10021, USA; postowm@mskcc.org; 6 Parker Institute for Cancer Immunotherapy, San Francisco, CA 94129, USA; 7 Medical Oncology, Dana-Farber Cancer Institute, Boston, MA 02115, USA; stephen_hodi@dfci.harvard.edu; 8 Medical Oncology, Royal Marsden NHS Foundation Trust, London SW3 6JJ, UK; james.larkin@rmh.nhs.uk; 9 Melanoma Cancer Immunotherapy and Innovative Therapy Unit, Istituto Nazionale Tumori IRCCS Fondazione Pascale, 80131 Naples, Italy; p.ascierto@istitutotumori.na.it; 10 Division of Cancer Services, Princess Alexandra Hospital, Woolloongabba, QLD 4102, Australia; victoria_atkinson@health.qld.gov.au; 11 Gallipoli Medical Research Foundation, Greenslopes Private Hospital, Greenslopes, QLD 4120, Australia; 12 Dermatology Service, Gustave Roussy, 94805 Villejuif, France; caroline.robert@gustaveroussy.fr; 13 Melanoma Research Unit, Paris-Saclay University, 91400 Orsay, France; 14 Melanoma Service, Memorial Sloan Kettering Cancer Center, New York, NY 10065, USA; 15 Bristol Myers Squibb, Princeton, NJ 08543, USA; sandra.re@bms.com (S.R.); david.paulucci@bms.com (D.P.); darin.dobler@bms.com (D.D.); 16 Precision Oncology Center, Centre Hospitalier Universitaire Vaudois (CHUV), 1011 Lausanne, Switzerland; olivier.michielin@chuv.ch

**Keywords:** proton pump inhibitors, checkpoint inhibitors, melanoma, pooled analysis

## Abstract

**Simple Summary:**

Immune checkpoint inhibitors have been shown to improve survival in patients with advanced melanoma; however, a proportion of patients do not experience durable clinical benefit with these agents. Findings from a previous study suggested that the use of proton pump inhibitors while receiving immune checkpoint inhibitors may lead to worse clinical outcomes. To validate those results, we performed this retrospective analysis using data from three clinical trials involving patients with advanced melanoma treated with immune checkpoint inhibitors. We found that there is not enough evidence to conclude that proton pump inhibitors influence the efficacy of immune checkpoint inhibitors. Prospective studies are needed to conclusively determine if the use of proton pump inhibitors has any meaningful impact on the efficacy of immune checkpoint inhibitors in patients with advanced melanoma.

**Abstract:**

The impact of proton pump inhibitors (PPIs) on clinical outcomes with first-line immune checkpoint inhibitors (ICIs) in patients with metastatic melanoma was previously analyzed in the phase II study, CheckMate 069. This retrospective analysis utilized data from three phase II/III studies of first-line ICI therapy in untreated advanced melanoma: CheckMate 066, 067, and 069. All randomized patients with PPI use ≤ 30 days before initiating study treatment were included in the PPI-use subgroup. Possible associations between baseline PPI use and efficacy were evaluated within each treatment arm of each study using multivariable modeling. Approximately 20% of 1505 randomized patients across the studies reported baseline PPI use. The median follow-up was 52.6–58.5 months. Objective response rate (ORR), progression-free survival (PFS), and overall survival analyses provided insufficient evidence of a meaningful association between PPI use and efficacy outcomes with nivolumab-plus-ipilimumab, nivolumab, or ipilimumab therapy. In five of the six ICI treatment arms, 95% confidence intervals for odds ratios or hazard ratios traversed 1. Significant associations were observed in the CheckMate 069 combination arm between PPI use and poorer ORR and PFS. This multivariable analysis found insufficient evidence to support meaningful associations between PPI use and ICI efficacy in patients with advanced melanoma.

## 1. Introduction

Immune checkpoint inhibitors (ICIs), such as the programmed death 1 (PD-1) inhibitor nivolumab, are well established as a treatment for patients with advanced melanoma [1], either alone or in combination with ipilimumab (a cytotoxic T-lymphocyte-associated protein 4 inhibitor), with durable survival rates demonstrated in randomized controlled phase III trials [2,3]. In the phase III CheckMate 066 trial, a 5-year overall survival (OS) rate of 39% was reported with nivolumab monotherapy in treatment-naive patients with wild-type *BRAF* advanced melanoma [3]. In the phase III CheckMate 067 trial, 5-year OS rates of 52% and 44% were demonstrated with nivolumab with or without ipilimumab, respectively, in previously untreated patients with advanced melanoma [2]. Despite these positive results in a disease that historically was regarded as having a very poor outcome, a proportion of patients with advanced melanoma do not experience durable clinical benefit with these agents [2,3].

The underlying reasons for heterogeneous responses to ICIs are not well understood. Indeed, reliable predictors of clinical benefits of these agents have yet to be identified, and approaches for overcoming innate and acquired tumor resistance to ICI therapies have yet to be developed [1]. Many factors may adversely affect ICI efficacy, such as the patient’s immune status [4,5,6,7,8] or co-administration of drugs, such as antibiotics, that reduce the diversity of the gut microbiome [7,8,9,10]. Both preclinical and clinical data suggest an association between the microbiome and the activity of ICIs against melanoma [7,8,11]. 

Proton pump inhibitors (PPIs) have also been shown to have an adverse effect on the gut microbiome [12,13]. A possible mechanism for this effect is the direct impact of PPIs on gastric pH, which is a major barrier to pathogens invading the GI tract. An effect of PPIs on the functionality of the immune system was also suggested in a study that found an increased risk of developing community-acquired pneumonia with PPI use [14].

As part of an effort to evaluate the impact of concomitant medications on clinical outcomes with ICIs, a previously presented retrospective analysis found that the use of PPIs reduced the efficacy of nivolumab and ipilimumab combination therapy but not ipilimumab alone, in 140 treatment-naive patients with metastatic melanoma [15]. That analysis utilized data from a randomized phase II study, CheckMate 069 [16,17]. Additionally, two large pooled retrospective analyses evaluated the impact of PPIs specifically on outcomes with ICIs using phase II/III trial data: one in non-small-cell lung cancer (NSCLC; *n* = 1512) and one in urothelial cancer (*n* = 1360) [10,18]. Both found that PPI use was associated with poor outcomes with ICIs. In order to validate the prior analysis of CheckMate 069 and evaluate the prognostic and predictive ability of PPI use in greater detail, in this study, we performed a retrospective analysis of the impact of PPI use on ICI outcomes across a total of 1505 treatment-naive patients with metastatic melanoma enrolled in CheckMate 066 and CheckMate 067, as well as CheckMate 069.

## 2. Materials and Methods

### 2.1. Patients, Study Design, and Treatment

This post hoc analysis utilized data from three multicenter, double-blinded, randomized studies of treatment-naive patients with advanced melanoma who had received ICI therapy: CheckMate 066, CheckMate 067, and CheckMate 069. Patient populations, study designs, and treatment regimens used in these studies have been described extensively [2,3,16,17,19,20,21,22,23] and are summarized in Table 1. All three studies were conducted in accordance with the provisions of the Declaration of Helsinki and the International Conference on Harmonisation Guidelines for Good Clinical Practice. All of the patients provided written informed consent.

### 2.2. Assessments

Tumor response was assessed on the schedules shown in Table 1 using Response Evaluation Criteria in Solid Tumors (RECIST), version 1.1. Objective response rate (ORR) per investigator was defined as the proportion of patients with a best overall response of partial or complete response, while progression-free survival (PFS) per investigator was defined as the time from randomization to first documented disease progression or death (whichever occurred first), and overall survival (OS) as the time from randomization to death.

### 2.3. Statistical Analysis

The aim of the statistical analysis was to evaluate the prognostic and predictive ability of baseline PPI use in patients who participated in CheckMate 066, 067, or 069. All analyses, which involved all randomized patients from each study, were performed based on separate database locks for each study. Baseline PPI use was determined using data entered on case report forms. The “PPI use” subgroup included patients who used any medication classified as a PPI (omeprazole, esomeprazole, pantoprazole, lansoprazole, rabeprazole, or dexlansoprazole) ≤ 30 days before the start of the study treatment. The “no PPI use” subgroup was defined as all other patients. The analysis did not consider PPI dosage, PPI treatment duration, or whether the patient continued to take a PPI during the study.

The association between PPI use at baseline and ORR, PFS, and OS was evaluated within each treatment arm of each study using multivariable modeling. Each model included PPI use at screening as a covariate in addition to a number of prognostic variables (i.e., age, sex, geographic region, race, Eastern Cooperative Oncology Group performance status [ECOG PS], metastasis stage, American Joint Committee on Cancer stage, history of brain metastases, PD-L1 status, lactate dehydrogenase level, and *BRAF* mutation status), which were based on clinical data from of each of the three studies; the particular prognostic variables utilized as covariates for each model varied between the studies and between efficacy outcomes (Supplementary Tables S1–S3). The analyses of ORR were performed using multivariable logistic regression models, in which odds ratios and corresponding two-sided 95% confidence intervals (CIs) were determined for all covariates in the model. The analyses of PFS and OS were performed using unstratified multivariable Cox proportional-hazards regression models, in which hazard ratios (HRs) and two-sided 95% CIs were determined for each covariate. In addition, PFS and OS distributions were estimated using the Kaplan–Meier method. Analyses were performed using SAS 9.2 software (SAS Institute, Cary, NC, USA).

## 3. Results

The majority of the 1505 patients across CheckMate 066, 067, and 069 had melanoma stage IV, M1c, *BRAF* wild-type disease, and no history of brain metastases (Table 2). Approximately 20% of patients had used PPIs at baseline across all three studies, and higher proportions of these patients were at least 75 years of age and/or had a baseline ECOG PS of 1 or higher than patients who had not used PPIs at baseline. Slight imbalances were also seen in other parameters, such as baseline lactose dehydrogenase levels, which tended to be higher in patients on PPIs. Median follow-up, derived as median time from randomization to database lock, was 58.5 months in CheckMate 066, 53.0 months in CheckMate 067, and 52.6 months in CheckMate 069.

**Figure 1:**
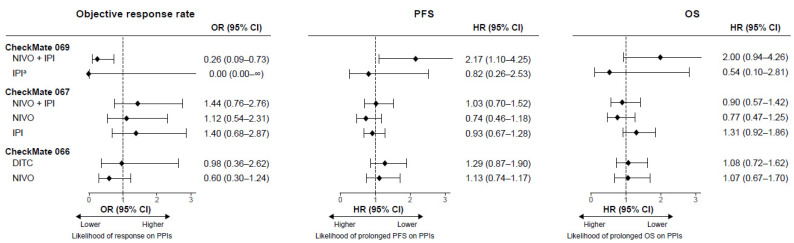
Association between baseline PPI use and efficacy in CheckMate 066, 067, and 069. Forest plot of ORs for objective response rate and HRs for OS and PFS derived from multivariable models. Error bars indicate 95% CIs. Point estimates and CIs for the association between PPI use (yes vs. no) and each outcome for all covariates included in the multivariable models are shown in Supplementary Tables S1–S3. ^a^ The logistic regression model for the objective response rate failed to converge in this group. CI: confidence interval; DITC: dacarbazine; HR: hazard ratio; IPI: ipilimumab; NIVO: nivolumab; OR: odds ratio; OS: overall survival; PFS: progression-free survival; PPI: proton pump inhibitor.

The overall results of the multivariable analyses of efficacy outcomes performed on each treatment group in each of the three studies are summarized in Figure 1. Detailed results showing the covariates included to control for confounding in the models are provided in Supplementary Tables S1–S3. Collectively, these results indicated that there is no evidence of a meaningful association between PPI use and outcomes with ICI therapy. In five of the six treatment arms involving ICIs in the three studies, the 95% CIs for odds ratios or HRs traversed 1. Significant associations were observed only in the nivolumab plus ipilimumab arm of CheckMate 069: between PPI use and poorer ORR and PFS outcomes as reported previously [15], with a trend toward poorer OS outcomes as well. Only seven patients in the ipilimumab arm of CheckMate 069 were on a PPI at baseline, resulting in multivariable analysis results for this arm that did not converge for ORR.

**Figure 2:**
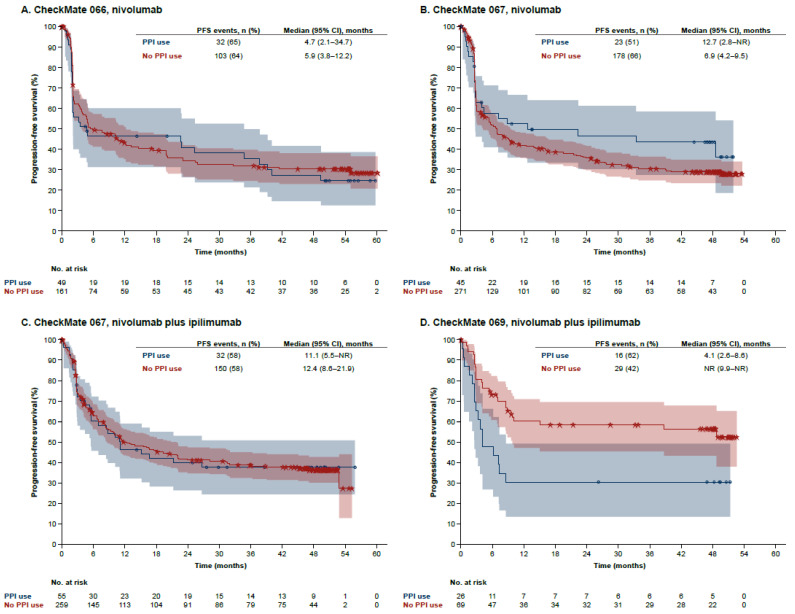
Progression-free survival by baseline PPI use in the nivolumab-containing treatment arms of CheckMate 066, 067, and 069. Kaplan–Meier estimates of progression-free survival are shown in the nivolumab arms of CheckMate 066 (**A**) and CheckMate 067 (**B**) and in the nivolumab plus ipilimumab arms of CheckMate 067 (**C**) and CheckMate 069 (**D**). Shaded areas are 95% log–log confidence bands. CI: confidence interval; PFS: progression-free survival; PPI: proton pump inhibitor.

Unadjusted results of Kaplan–Meier analyses of PFS by PPI use in the nivolumab-containing arms of the three studies are shown in Figure 2. In each of the treatment arms of CheckMate 066 and 067, PFS curves by PPI usage did not separate, although a trend toward improved PFS with PPI use was observed in the nivolumab arm of CheckMate 067. This trend was not confirmed in multivariable analysis after adjustment for other prognostic variables (Supplementary Table S2). In CheckMate 069, however, reduced PFS was observed with PPI usage in the nivolumab plus ipilimumab arm, which was confirmed in the multivariable analyses. Kaplan–Meier analysis of the dacarbazine and ipilimumab arms of the studies showed no discernible effect of PPI use on PFS (Supplementary Figure S1).

**Figure 3:**
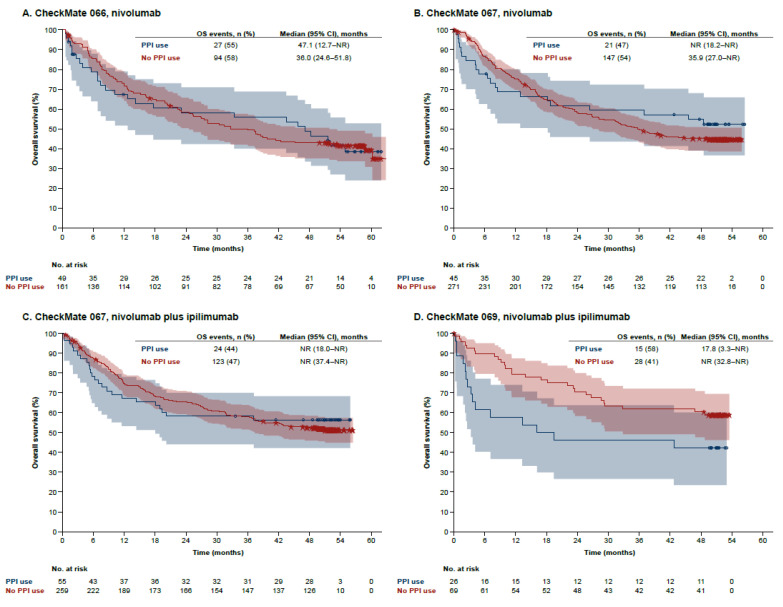
Overall survival by baseline PPI use in the nivolumab-containing treatment arms of CheckMate 066, 067, and 069. Kaplan–Meier estimates of overall survival are shown in the nivolumab arms of CheckMate 066 (**A**) and CheckMate 067 (**B**) and in the nivolumab plus ipilimumab arms of CheckMate 067 (**C**) and CheckMate 069 (**D**). Shaded areas are 95% log–log confidence bands. CI: confidence interval; OS: overall survival; PPI: proton pump inhibitor.

Univariate Kaplan–Meier analysis similarly showed no association between PPI use and OS with nivolumab in CheckMate 066, a possible association (small and negative) with nivolumab and nivolumab plus ipilimumab treatment in CheckMate 067, and a more pronounced negative association with nivolumab plus ipilimumab treatment in CheckMate 069 (Figure 3). Multivariate analysis showed that there was no significant evidence for associations in any of the studies, although a directional trend for poorer OS with nivolumab plus ipilimumab in CheckMate 069 was observed (Supplementary Table S3). Trends toward associations between PPI use and poorer OS in the dacarbazine arm of CheckMate 066 and the ipilimumab arm of CheckMate 067 suggested by Kaplan–Meier analysis (Supplementary Figure S2) were not supported after adjustment for other variables in the multivariate analyses (Supplementary Table S3).

## 4. Discussion

The collective results of multivariable analyses of CheckMate 066, 067, and 069 involving a total of 1505 patients did not support a meaningful association between baseline PPI use and the efficacy of ICI treatment in patients with advanced melanoma. No significant associations were observed with any study drugs in the large global phase III studies CheckMate 066 (nivolumab or dacarbazine) or CheckMate 067 (nivolumab alone, nivolumab plus ipilimumab, or ipilimumab alone). However, PPI use by patients treated with nivolumab plus ipilimumab in the phase II CheckMate 069 study was associated with reduced ORR and PFS, with a directional trend observed for OS.

The negative association between PPI use and outcomes with ICIs found in CheckMate 069 in the present analysis confirms similar observations made by several of us in an earlier analysis in which PPIs were one of a number of concomitant medications that were evaluated [15]. These results differed substantially from those of CheckMate 066 and 067, however, and the precise reasons for this discrepancy are unclear. CheckMate 069 was carried out predominantly in the United States with some enrollment in France, while CheckMate 066 and 067 were global studies. Consistent with the diverse geographic distributions of the three studies, some characteristics of the study populations differed between the trials. Compared with patient populations in CheckMate 066 and 067, patients in CheckMate 069 tended to have better prognostic characteristics: proportions of patients with an ECOG PS of 0 or PD-L1–positive tumors were higher, and those with elevated levels of LDH or M1c disease were lower. A case–control analysis of data from matched patients in CheckMate 067 and 069 might potentially shed light on relevant prognostic factors, although this would be hypothesis-generating only. Additionally, of note is that, within the current analysis, the patient populations differed with respect to *BRAF* mutation status (a known prognostic factor in melanoma): CheckMate 066 enrolled patients with *BRAF* wild-type disease, whereas CheckMate 067 and 069 enrolled patients with both mutant and wild-type disease. However, with only 46 patients across CheckMate 067 and 069 having *BRAF* mutant melanoma, the data are insufficient to evaluate the impact of PPI use on ICI efficacy based on *BRAF* mutation status. Prospective studies are needed to conclusively determine the impacts of baseline prognostic characteristics on outcomes with ICIs in patients reporting PPI use.

A number of studies have examined the impact of PPIs on outcomes with ICI therapy in patients with various cancers outside of the present study; however, all were retrospective and involved patients with advanced disease [10,18,27,28,29,30,31,32,33,34,35,36]. Overall, the results were equivocal. The two large pooled retrospective analyses of phase II/III studies in NSCLC and urothelial cancer described earlier [10,18] found that PPI use was associated with poor outcomes with ICIs. A large, real-world, multicenter chart review of patients with NSCLC likewise found a negative impact of PPI use on clinical outcomes with pembrolizumab but also noted an association between PPI use and higher baseline ECOG PS, as was also seen in the current analysis [36]. The remainder of the studies were retrospective single-center chart reviews, each involving approximately 100 to 200 patients with a variety of advanced cancers, the majority of which found no association between PPI use and clinical outcomes with ICIs [29,30,32,33,34,35]. Several single-center chart reviews that focused on advanced melanoma found a favorable effect of PPI use on outcomes with ICIs in two cases and an unfavorable effect in one case [27,28,31]. Recent reviews of this subject were unable to make robust recommendations about PPI usage in patients being treated with ICIs, given the inconsistent data available from these studies, many of which were underpowered [37,38]. Our large analysis is consistent with both a lack of association between PPI use and ICI outcomes and the heterogeneity of the results obtained between studies. However, differences in trial design between the present study and previous analyses preclude broad conclusions from being made. For example, differences in the immune checkpoint inhibitors evaluated (e.g., anti–PD-1 monotherapy and combination of anti–PD-1 and anti–CTLA-4 therapy), particularly when comparing studies across tumor types, complicate the ability to generalize the results of these studies.

PPIs are selective inhibitors of H^+^/K^+^ ATPases, and they have multiple effects on the gastrointestinal (GI) microbiome [13]. PPIs reduce the diversity of GI microbiota and select for Lactobacilli, especially Streptococcaceae, in the upper GI tract. These pharyngeal commensals are able to move to the lower GI tract because PPIs disrupt the natural gastric acid barrier between the upper and lower GI tract. Although a causal link between aberrations in the GI microbiome, and the efficacy of immune checkpoint inhibitors has not yet been demonstrated, patients with advanced melanoma who have highly diverse GI microbiomes have superior systemic and antitumor immunological responses, compared with those in patients with low-diversity GI microbiomes [7,8]. The negative impact of PPIs on antitumor therapy observed in several studies has been suggested to be due to effects on the GI microbiome [29,30,31,32,33,35,39,40]. It has also been suggested that responses to ICIs can be negatively or positively influenced by the composition of the GI microbiome, which could explain the heterogeneous results observed in the studies cited above [8,9,11,37,41]. Unfortunately, data regarding patients’ microbiome profiles were not available for the current analysis within the scope of this manuscript.

The present analysis has some additional inherent limitations. It was an exploratory post hoc analysis among three studies differing in geographic location, design, and patient populations. As this analysis of patient subgroups based on PPI usage was not prespecified, some patient subgroups were too small for meaningful comparisons (e.g., the subgroup of patients who used PPIs in the ipilimumab arm of CheckMate 069 (*n* = 7). Additionally, PPI use was determined retrospectively using self-reported information from the prior medication pages of the patient case report form. The assumption that the absence of self-reported PPI use denotes the absence of PPI use at baseline carries the risk that a patient could be misclassified into the “no PPI use” subgroup due to a failure to report. Furthermore, the potential effects of PPI dosage, treatment duration, and discontinuation were not evaluated in this study. We also acknowledge that significant findings in the present post hoc analysis should be interpreted with caution, given that the relatively small sample sizes of patients reporting PPI use may have magnified differences in efficacy. This limitation is particularly relevant when considering outcomes observed in the “PPI use” subgroup of the phase II CheckMate 069 study (*n* = 33), which was substantially smaller than the corresponding subgroups of the phase III CheckMate 066 (*n* = 97) and 067 (*n* = 161) studies.

## 5. Conclusions

In conclusion, the results of our large multivariable analysis of three phase II/III studies found insufficient evidence to support a meaningful association between PPI use and efficacy outcomes with ICI therapy in patients with advanced melanoma. Ultimately, prospective studies will be needed to conclusively determine the impact of PPI usage on ICI efficacy in patients with melanoma or other cancers.

## Figures and Tables

**Table 1:** Trial overview.

Trial Characteristic	CheckMate 066 [3,19,20,24]	CheckMate 067 [2,21,22,23,25]	CheckMate 069 [16,17,26]
ClinicalTrials.gov no.	NCT01721772	NCT01844505	NCT01927419
Study phase	III	III	II
Study design	Multicenter, randomized, double-blind	Multicenter, randomized, double-blind	Multicenter, randomized, double-blind
Key eligibility criteria	Unresected stage III/IV melanoma Previously untreated *BRAF* wild-type ECOG PS 0/1	Unresected stage III/IV melanoma Previously untreated *BRAF* wild-type or mutant ECOG PS 0/1	Unresected stage III/IV melanoma Previously untreated *BRAF* wild-type or mutant ECOG PS 0/1
Randomization	1:1	1:1:1	2:1
Treatment groups	Nivolumab 3 mg/kg Q2W (*n* = 210) Dacarbazine 1000 mg/m^2^ Q3W (*n* = 208)	Nivolumab 1 mg/kg + ipilimumab 3 mg/kg Q3W × 4 → nivolumab 3 mg/kg Q2W (*n* = 314) Nivolumab 3 mg/kg Q2W (*n* = 316) Ipilimumab 3 mg/kg Q3W × 4 (*n* = 315)	Nivolumab 1 mg/kg + ipilimumab 3 mg/kg Q3W × 4 → nivolumab 3 mg/kg Q2W (*n* = 95) Ipilimumab 3 mg/kg Q3W × 4 (*n* = 47)
Primary endpoint(s)	OS	PFS OS	ORR in *BRAF* wild-type population
Secondary/exploratory endpoints ^a^	PFS ORR OS by PD-L1 expression Safety HRQoL	ORR Efficacy by PD-L1 expression Safety HRQoL	PFS in *BRAF* wild-type population ORR in *BRAF* mutant population PFS in *BRAF* mutant population HRQoL OS Safety
Tumor assessment	9 weeks after randomization Then Q6W through year 1 Then Q12W until PD/treatment discontinuation	12 weeks after randomization Then Q6W for 49 weeks Then Q12W until PD/treatment discontinuation	12 weeks after first treatment Then Q6W through year 1 Then Q12W until PD/treatment discontinuation
Study period	2013–2021	2013–ongoing	2013–2021

^a^ Key; not a comprehensive list. →: followed by; ECOG PS: Eastern Cooperative Oncology Group performance status; HRQoL: health-related quality of life; ORR: objective response rate; OS: overall survival; PD: progressive disease; PD-L1: programmed death-ligand 1; PFS: progression-free survival; Q2W: every 2 weeks; Q3W: every 3 weeks; Q6W: every 6 weeks; Q12W: every 12 weeks.

**Table 2:** Baseline characteristics by PPI usage in CheckMate 066, 067, and 069.

Baseline Characteristic	CheckMate 066 (*n* = 418)		CheckMate 067 (*n* = 945)		CheckMate 069 (*n* = 142)	
	PPI Use	No PPI Use	PPI Use	No PPI Use	PPI Use	No PPI Use
**PPI use status ^a^**	97 (23)	321 (77)	161 (17)	784 (83)	33 (23)	109 (77)
**Age (y)**						
<65	36 (37)	163 (51)	74 (46)	491 (63)	16 (48)	52 (48)
≥65–<75	38 (39)	114 (36)	55 (34)	207 (26)	12 (36)	45 (41)
≥75	23 (24)	44 (14)	32 (20)	86 (11)	5 (15)	12 (11)
**Sex**						
Female	45 (46)	127 (40)	52 (32)	283 (36)	11 (33)	36 (33)
Male	52 (54)	194 (60)	109 (68)	501 (64)	22 (67)	73 (67)
**Region**						
Western Europe/ Canada	62 (64)	228 (71)	-	-	-	-
US	-	-	31 (19)	176 (22)	29 (88)	97 (89)
France	-	-	-	-	4 (12)	12 (11)
EU	-	-	86 (53)	431 (55)	-	-
Australia	-	-	25 (16)	90 (11)	-	-
Rest of world	35 (36)	93 (29)	19 (12)	87 (11)	-	-
**Race**						
White	97 (100)	319 (99)	157 (98)	764 (97)	32 (97)	107 (98)
Asian	0	1 (<1)	0	10 (1)	1 (3)	0
Other	0	1 (<1)	4 (2)	10 (1) ^b^	0	2 (2)
**ECOG PS**						
0	41 (42)	228 (71)	97 (60)	594 (76)	22 (67)	94 (86)
≥1 ^c^	56 (58)	92 (29)	64 (40)	189 (24)	11 (33)	15 (14)
Missing	0	1 (<1)	0	1 (<1)	0	0
**M stage**						
M0	6 (6)	22 (7)	5 (3)	42 (5)	3 (9)	10 (9)
M1a	6 (6)	37 (12)	21 (13)	113 (14)	2 (6)	21 (19)
M1b	22 (23)	72 (22)	36 (22)	171 (22)	10 (30)	29 (27)
M1c	63 (65)	190 (59)	99 (61)	458 (59)	17 (52)	48 (44)
Not reported	0	0	0	0	1 (3)	1 (1)
**AJCC stage**						
III	12 (12)	37 (12)	8 (5)	55 (7)	4 (12)	15 (14)
IV	85 (88)	284 (88)	153 (95)	729 (93)	29 (88)	94 (86)
**History of brain metastases**						
No	94 (97)	309 (96)	152 (94)	760 (97)	33 (100)	104 (95)
Yes	3 (3)	12 (4)	9 (6)	24 (3)	0	4 (4)
Not reported	0	0	0	0	0	1 (1)
**PD-L1 status**						
Indeterminate/ negative	50 (52)	157 (49)	76 (47)	379 (48)	13 (39)	61 (56)
Positive	47 (48)	164 (51)	85 (53)	405 (52)	20 (61)	48 (44)
**LDH**						
≤ULN	44 (45)	200 (62)	97 (60)	493 (63)	23 (70)	83 (76)
>ULN	47 (48)	106 (33)	62 (39)	279 (36)	10 (30)	25 (23)
Not reported/ missing	6 (6)	15 (5)	2 (1)	12 (2)	0	1 (1)
***BRAF* status**						
Wild-type	96 (99)	314 (98)	123 (76)	521 (66)	25 (76)	85 (78)
Mutant	0	0	38 (24)	263 (34)	8 (24)	24 (22)
Missing ^d^	1 (1)	7 (2)	0	0	0	0

Data are n (%). ^a^ Numbers of patients in this row represent the evaluable population for all subsequent percentages in the corresponding column. ^b^ Includes one patient with missing data. ^c^ Four patients in CheckMate 066, one in CheckMate 067, and two in CheckMate 069 were enrolled in each study despite having an ECOG PS of 2 [16,19,21]. ^d^ Two patients in the decarbazine arm of CheckMate 066 tested positive for *BRAF* V600 mutation following post-study biopsies [3]. AJCC: American Joint Committee on Cancer; ECOG PS: Eastern Cooperative Oncology Group performance status; EU: European Union; M stage: metastasis stage; LDH: lactate dehydrogenase; PD-L1: programmed death-ligand 1; PPI: proton pump inhibitor; ULN: upper limit of normal.

## Data Availability

Bristol Myers Squibb’s policy on data sharing may be found at https://www.bms.com/researchers-and-partners/independent-research/data-sharing-request-process.html (accessed on 27 March 2022).

## Supplementary Materials

The following supporting information can be downloaded at: https://www.mdpi.com/article/10.3390/cancers14092300/s1, Table S1: Multivariable logistic regression models of ORR by study and treatment, Table S2: Multivariable Cox proportional-hazards models of PFS by study and treatment, Table S3: Multivariable Cox proportional-hazards models of OS by study and treatment, Figure S1: Progression-free survival by baseline PPI use in the control arms of CheckMate 066, 067, and 069, Figure S2: Overall survival by baseline PPI use in the control arms of CheckMate 066, 067, and 069.
