# Supplementary material for: Proton Pump Inhibitor Use and Efficacy of Nivolumab and Ipilimumab in Advanced Melanoma
Source: Cancers (Basel). 2022 May 5;14(9):2300. doi: 10.3390/cancers14092300 (PMC9103038; doi:10.3390/cancers14092300)
Supplement: Supplementary file 1 [file cancers-14-02300-s001.zip › cancers-1687548-supplementary.pdf]

cancers

MDPI

Supplementary Materials

Proton Pump Inhibitor Use and Efficacy of Nivolumab and Ipilimumab in Advanced Melanoma

Krisztian Homicsko [1,*], Reinhard Dummer [2], Christoph Hoeller [3], Jedd D. Wolchok [4,5,6], F. Stephen Hodi [7], James Larkin [8], Paolo A. Ascierto [9], Victoria Atkinson [10,11], Caroline Robert [12,13], Michael A. Postow [5,14], Sandra Re [15], David Paulucci [15], Darin Dobler [15] and Olivier Michielin [16]

1 Department of Oncology, Centre Hospitalier Universitaire Vaudois (CHUV), 1011 Lausanne, Switzerland
2 Department of Dermatology, Universitäts Spital, 8091 Zurich, Switzerland; reinhard.dummer@usz.ch
3 Department of Dermatology, Medical University of Vienna, 1090 Vienna, Austria; christoph.hoeller@meduniwien.ac.at
4 Human Oncology and Pathogenesis Program, Memorial Sloan Kettering Cancer Center, New York, NY 10065, USA; wolchokj@mskcc.org
5 Department of Medicine, Weill Cornell Medical College, New York, NY 10021, USA; postowm@mskcc.org
6 Parker Institute for Cancer Immunotherapy, San Francisco, CA 94129, USA
7 Medical Oncology, Dana-Farber Cancer Institute, Boston, MA 02115, USA; stephen_hodi@dfci.harvard.edu
8 Medical Oncology, Royal Marsden NHS Foundation Trust, London SW3 6JJ, UK; james.larkin@rmh.nhs.uk
9 Melanoma Cancer Immunotherapy and Innovative Therapy Unit, Istituto Nazionale Tumori IRCCS Fondazione Pascale, 80131 Naples, Italy; p.ascierto@istitutotumori.na.it
10 Division of Cancer Services, Princess Alexandra Hospital, Woolloongabba, QLD 4102, Australia; victoria_atkinson@health.qld.gov.au
11 Gallipoli Medical Research Foundation, Greenslopes Private Hospital, Greenslopes, QLD 4120, Australia
12 Dermatology Service, Gustave Roussy, 94805 Villejuif, France; caroline.robert@gustaveroussy.fr
13 Melanoma Research Unit, Paris-Saclay University, 91400 Orsay, France
14 Melanoma Service, Memorial Sloan Kettering Cancer Center New York, NY 10065, USA
15 Bristol Myers Squibb, Princeton, NJ 08543, USA; sandra.re@bms.com (S.R.); david.paulucci@bms.com (D.P.); darin.dobler@bms.com (D.D.)
16 Precision Oncology Center, Centre Hospitalier Universitaire Vaudois (CHUV), 1011 Lausanne, Switzerland; olivier.michielin@chuv.ch
* Correspondence: krisztian.homicsko@chuv.ch

Table S1. Multivariable logistic regression models of ORR by study and treatment.

Parameter	CheckMate 066		CheckMate 067			CheckMate 069	
	Dacarbazine (*N* = 198)	Nivolumab (*N* = 199)	Ipilimumab (*N* = 309)	Nivolumab (*N* = 309)	Nivolumab + Ipilimumab (*N* = 313)	Ipilimumab (*N* = 46)	Nivolumab + Ipilimumab (*N* = 93)
PPI at baseline (yes vs no)	0.975 (0.362-2.625) [0.96]	0.605 (0.295-1.239) [0.17]	1.395 (0.678-2.866) [0.37]	1.116 (0.539-2.310) [0.77]	1.444 (0.756-2.756) [0.27]	0.000 (0.000-infinity) [0.97]	0.256 (0.090-0.731) [0.01]
Metastatic disease stage (M1c vs M0/M1a/M1b)	0.682 (0.280-1.661) [0.40]	0.790 (0.407-1.533) [0.49]	0.722 (0.384-1.358) [0.31]	0.967 (0.561-1.667) [0.90]	0.624 (0.317-1.050) [0.08]	189045 (0.000-infinity) [0.93]	0.997 (0.381-2.606) [0.99]
Sex (male vs female)	0.395 (0.173-0.904) [0.03]	1.437 (0.782-2.642) [0.24]	1.490 (0.775-2.864) [0.23]	1.960 (1.155-3.327) [0.01]	1.427 (0.864-2.357) [0.16]	107150 (0.000-infinity) [0.94]	1.955 (0.751-5.087) [0.17]
LDH (> ULN vs ≤ ULN)	0.877 (0.341-2.252) [0.78]	0.572 (0.300-1.093) [0.09]	0.349 (0.168-0.723) [0.005]	0.487 (0.277-0.856) [0.01]	0.466 (0.280-0.777) [0.003]	0.258 (0.018-3.618) [0.31]	0.507 (0.182-1.412) [0.19]
AJCC disease stage (IV vs III)	0.289 (0.098-0.857) [0.03]	0.972 (0.392-2.409) [0.95]	0.517 (0.179-1.496) [0.22]	0.462 (0.182-1.169) [0.10]	0.684 (0.216-2.161) [0.52]	1.393 (0.000-infinity) [1.00]	2.359 (0.537-10.363) [0.26]
PD-L1–positive (≥ 1% vs indeterminate/negative)	0.981 (0.426-2.257) [0.96]	2.126 (1.178-3.840) [0.01]	1.058 (0.574-1.950) [0.86]	2.068 (1.240-3.448) [0.01]	1.479 (0.915-2.391) [0.11]	2.392 (0.218-26.201) [0.48]	1.922 (0.727-5.085) [0.19]

Region							
Rest of world vs western Europe/Canada	1.343 (0.572-3.152) [0.50]	1.242 (0.651-2.371) [0.51]	-	-	-	-	-
Rest of world vs US	-	-	0.486 (0.177-1.335) [0.16]	1.462 (0.618-3.457) [0.39]	0.684 (0.280-1.666) [0.40]	-	-
EU vs US	-	-	0.337 (0.169-0.672) [0.002]	0.783 (0.419-1.462) [0.44]	0.827 (0.447-1.530) [0.55]	-	-
Australia vs US	-	-	0.291 (0.096-0.877) [0.03]	3.092 (1.216-7.865) [0.02]	1.852 (0.741-4.632) [0.19]	-	-
France vs US	-	-	-	-	-	0.000 (0.000-infinity) [0.96]	2.000 (0.455-8.792) [0.36]

Data are odds ratio (95% CI) [P value]. AJCC, American Joint Committee on Cancer; EU, European Union; LDH, lactate dehydrogenase; PD-L1, programmed death-ligand 1; PPI, proton pump inhibitor; ULN, upper limit of normal; US, United States.

Table S2. Multivariable Cox proportional hazards models of PFS by study and treatment.

Parameter	**CheckMate 066**		**CheckMate 067**			**CheckMate 069**	
	**Dacarbazine (*N* = 198)**	**Nivolumab (*N* = 199)**	**Ipilimumab (*N* = 309)**	**Nivolumab (*N* = 309)**	**Nivolumab + Ipilimumab (*N* = 313)**	**Ipilimumab (*N* = 46)**	**Nivolumab + Ipilimumab (*N* = 93)**
PPI at baseline (yes vs no)	1.286 (0.869-1.903) [0.21]	1.126 (0.741-1.713) [0.58]	0.926 (0.669-1.282) [0.65]	0.741 (0.464-1.183) [0.21]	1.032 (0.700-1.520) [0.87]	0.818 (0.264-2.529) [0.73]	2.167 (1.105-4.251) [0.02]
History of brain metastases (yes vs no)	1.240 (0.556-2.767) [0.60]	1.269 (0.466-3.456) [0.64]	0.766 (0.395-1.486) [0.43]	0.846 (0.304-2.357) [0.75]	0.428 (0.134-1.363) [0.15]	NA NA NA	1.220 (0.262-5.680) [0.80]
Metastatic disease stage (M1c vs M0/M1a/M1b)	1.283 (0.906-1.817) [0.16]	1.107 (0.744-1.646) [0.62]	1.357 (1.043-1.766) [0.02]	1.230 (0.906-1.669 [0.18]	1.561 (1.127-2.163) [0.01]	0.568 (0.265-1.215) [0.15]	1.263 (0.676-2.362) [0.46]
Sex (male vs female)	1.430 (1.024-1.997) [0.04]	0.747 (0.515-1.084) [0.12]	1.079 (0.830-1.402) [0.57]	0.762 (0.566-1.024) [0.07]	0.798 (0.586-1.086) [0.15]	0.556 (0.250-1.237) [0.15]	0.638 (0.337-1.208) [0.17]
ECOG PS (≥ 1 vs 0)	1.095 (0.768-1.562) [0.62]	1.280 (0.839-1.951) [0.25]	1.189 (0.898-1.574) [0.23]	1.451 (1.019-2.065) [0.04]	1.295 (0.924-1.814) [0.13]	0.918 (0.313-2.696) [0.88]	1.239 (0.513-2.995) [0.63]
LDH (> ULN vs ≤ ULN)	1.703 (1.187-2.443) [0.004]	1.530 (1.007-2.325) [0.05]	2.044 (1.570-2.661) [0.0000]	1.468 (1.063-2.028) [0.02]	1.657 (1.208-2.273) [0.002]	2.316 (0.839-6.396) [0.11]	1.447 (0.713-2.936) [0.31]
PD-L1–positive (≥1% vs indeterminate/negative)	1.183 (0.856-1.635) [0.31]	0.609 (0.425-0.872) [0.01]	0.676 (0.524-0.873) [0.003]	0.663 (0.491-0.894) [0.01]	0.855 (0.637-1.150) [0.30]	1.059 (0.482-2.331) [0.89]	0.751 (0.394-1.430) [0.38]
Region							
Rest of world vs western Europe/Canada	0.671 (0.461-0.978) [0.04]	0.893 (0.586-1.363) [0.60]	-	-	-	-	-
Rest of world vs US	-	-	1.068 (0.678-1.681) [0.78]	0.780 (0.460-1.325) [0.36]	1.755 (1.027-2.999) [0.04]	-	-
EU vs US	-	-	1.368 (0.996-1.879) [0.05]	1.184 (0.813-1.723) [0.38]	1.332 (0.899-1.972) [0.15]	-	-

Australia vs US	-	-	1.334 (0.862-2.063) [0.20]	0.708 (0.403-1.245) [0.23]	0.639 (0.346-1.180) [0.15]	-	-
France vs US	-	-	-	-	-	0.762 (0.224-2.598) [0.66]	0.621 (0.187-2.062) [0.44]

Data are hazard ratio (95% CI) [P value]. AJCC, American Joint Committee on Cancer; ECOG PS, Eastern Cooperative Oncology Group performance status; EU, European Union; LDH, lactate dehydrogenase; NA, not applicable (no patients in the CheckMate 069 ipilimumab arm had brain metastases at baseline); PD-L1, programmed death-ligand 1; PPI, proton pump inhibitor; ULN, upper limit of normal; US, United States.

Table S3. Multivariable Cox proportional hazards models of OS by study and treatment.

Parameter	CheckMate 066		CheckMate 067			CheckMate 069	
	**Dacarbazine (*N* = 198)**	**Nivolumab (*N* = 199)**	**Ipilimumab (*N* = 309)**	**Nivolumab (*N* = 309)**	**Nivolumab + Ipilimumab (*N* = 313)**	**Ipilimumab (*N* = 46)**	**Nivolumab + Ipilimumab (*N* = 93)**
PPI at baseline (yes vs no)	1.077 (0.719-1.613) [0.72]	1.069 (0.673-1.697) [0.78]	1.309 (0.922-1.857) [0.13]	0.766 (0.468-1.254) [0.29]	0.903 (0.573-1.425) [0.66]	0.538 (0.103-2.811) [0.46]	1.996 (0.936-4.256) [0.07]
History of brain metastases (yes vs no)	0.784 (0.309-1.991) [0.61]	1.028 (0.336-3.140) [0.96]	0.824 (0.410-1.659) [0.59]	0.922 (0.331-2.569) [0.88]	0.777 (0.275-2.197) [0.63]	NA NA NA	2.919 (0.581-14.667) [0.19]
BRAF mutation status (mutant vs wild type)	-	-	0.971 (0.713-1.323) [0.85]	0.709 (0.493-1.019) [0.06]	0.661 (0.448-0.974) [0.04]	0.190 (0.044-0.829) [0.03]	2.594 (1.118-6.018) [0.03]
Metastatic disease stage (M1c vs M0/M1a/M1b)	1.599 (1.112-2.302) [0.01]	1.181 (0.773-1.805) [0.44]	1.667 (1.238-2.245) [0.001]	1.697 (1.195-2.410) [0.003]	1.671 (1.148-2.432) [0.007]	0.443 (0.155-1.266) [0.13]	1.575 (0.800-3.101) [0.19]
Age group (≥ 65–< 75 vs < 65)	1.017 (0.696-1.486) [0.93]	0.814 (0.534-1.242) [0.34]	0.785 (0.565-1.091) [0.15]	0.945 (0.644-1.387) [0.77]	1.194 (0.814-1.750) [0.36]	0.351 (0.118-1.047) [0.06]	1.730 (0.798-3.749) [0.16]
Age group (≥ 75 vs < 65)	1.798 (1.132-2.858) [0.01]	0.697 (0.366-1.328) [0.27]	1.252 (0.817-1.920) [0.30]	1.427 (0.893-2.280) [0.14]	1.256 (0.747-2.113) [0.39]	1.503 (0.380-5.936) [0.56]	3.733 (1.291-10.797) [0.02]
Sex (male vs female)	1.190 (0.839-1.688) [0.33]	0.681 (0.460-1.008) [0.05]	1.105 (0.826-1.479) [0.50]	0.799 (0.573-1.114) [0.19]	0.758 (0.537-1.070) [0.11]	1.156 (0.374-3.569) [0.80]	0.596 (0.303-1.171) [0.13]
ECOG PS (≥ 1 vs 0)	1.417 (0.989-2.031) [0.06]	2.225 (1.393-3.554) [0.001]	1.587 (1.168-2.157) [0.003]	1.936 (1.345-2.786) [0.0004]	2.079 (1.458-2.963) [0.0001]	2.957 (0.752-11.634) [0.12]	1.216 (0.515-2.866) [0.66]
LDH (> ULN vs ≤ ULN)	2.437 (1.668-3.562) [0.0000]	1.713 (1.115-2.632) [0.01]	2.134 (1.596-2.855) [0.0000]	1.494 (1.043-2.141) [0.03]	1.970 (1.385-2.801) [0.0002]	10.914 (2.823-42.197) [0.0005]	1.651 (0.708-3.851) [0.25]
PD-L1–positive (≥ 1% vs indeterminate/negative)	1.615 (1.151-2.266) [0.01]	0.647 (0.440-0.951) [0.03]	0.728 (0.549-0.965) [0.03]	0.671 (0.482-0.933) [0.02]	0.914 (0.654-1.278) [0.60]	0.765 (0.303-1.935) [0.57]	0.491 (0.238-1.013) [0.05]
Region							
Rest of world vs western Europe/Canada	0.914 (0.632, 1.322) [0.63]	0.732 (0.463, 1.155) [0.18]	-	-	-	-	-
Rest of world vs US	-	-	1.098 (0.655,1.840) [0.72]	0.936 (0.534,1.642) [0.82]	2.481 (1.344,4.580) [0.004]	-	-

EU vs US	-	-	1.366 (0.960-1.944) [0.08]	1.270 (0.846-1.907) [0.25]	1.609 (1.011-2.562) [0.04]	-	-
Australia vs US	-	-	0.801 (0.470-1.364) [0.41]	0.634 (0.335-1.201) [0.16]	0.716 (0.351-1.464) [0.36]	-	-
France vs US	-	-	-	-	-	0.114 (0.017-0.739) [0.02]	0.629 (0.211-1.873) [0.40]

Data are hazard ratio (95% CI) [P value]. AJCC, American Joint Committee on Cancer; ECOG PS, Eastern Cooperative Oncology Group performance status; EU, European Union; LDH, lactate dehydrogenase; NA, not applicable (no patients in the CheckMate 069 ipilimumab arm had brain metastases at baseline); PD-L1, programmed death-ligand 1; PPI, proton pump inhibitor; ULN, upper limit of normal; US, United States.

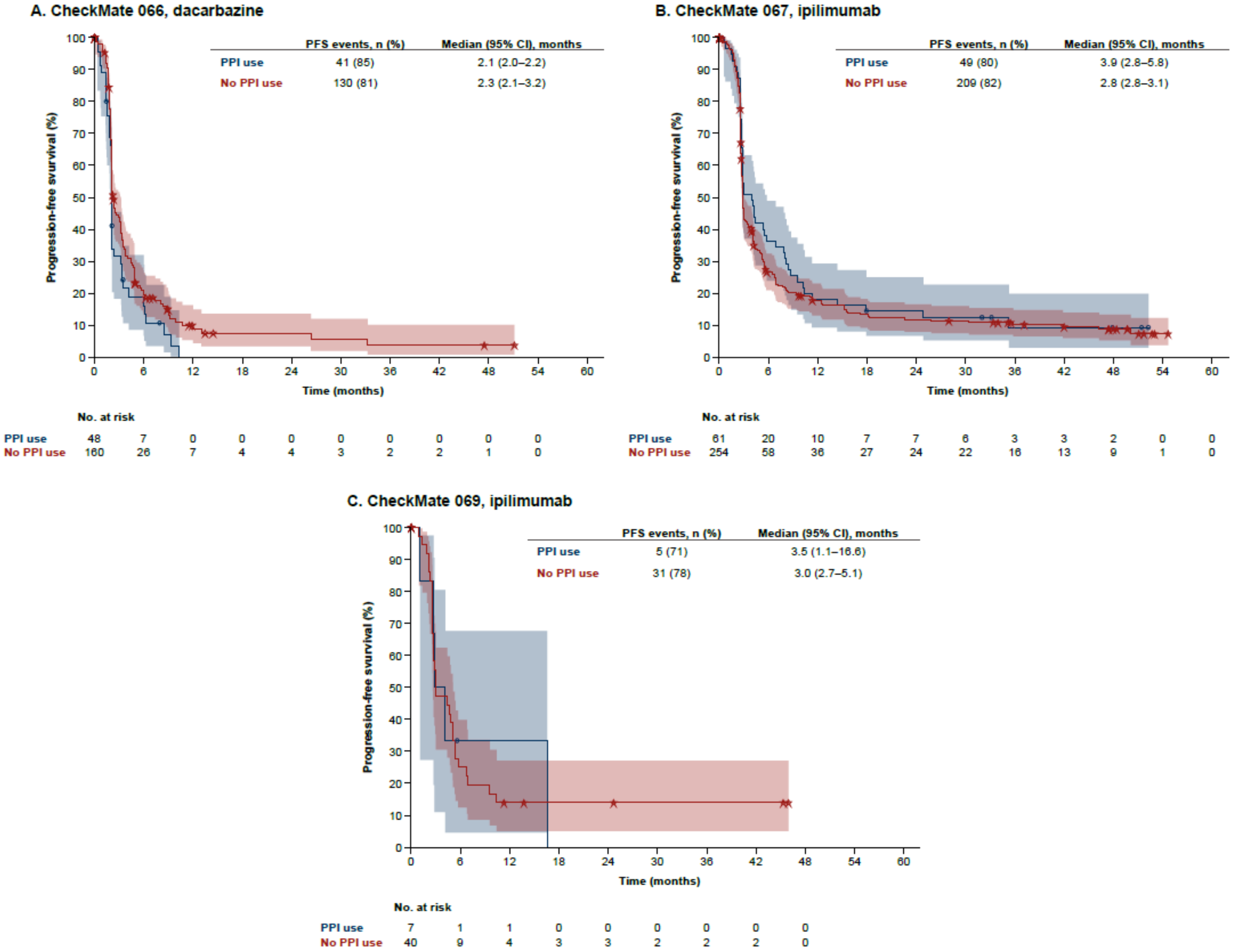

Figure S1. Progression-free survival by baseline PPI use in the control arms of CheckMate 066, 067, and 069. Kaplan–Meier estimates of progression-free survival in the dacarbazine arm of CheckMate 066 (**A**) and the ipilimumab arms of CheckMate 067 (**B**) and CheckMate 069 (**C**). Shaded areas are 95% log–log confidence bands. CI, confidence interval; PFS, progression-free survival; PPI, proton pump inhibitor.

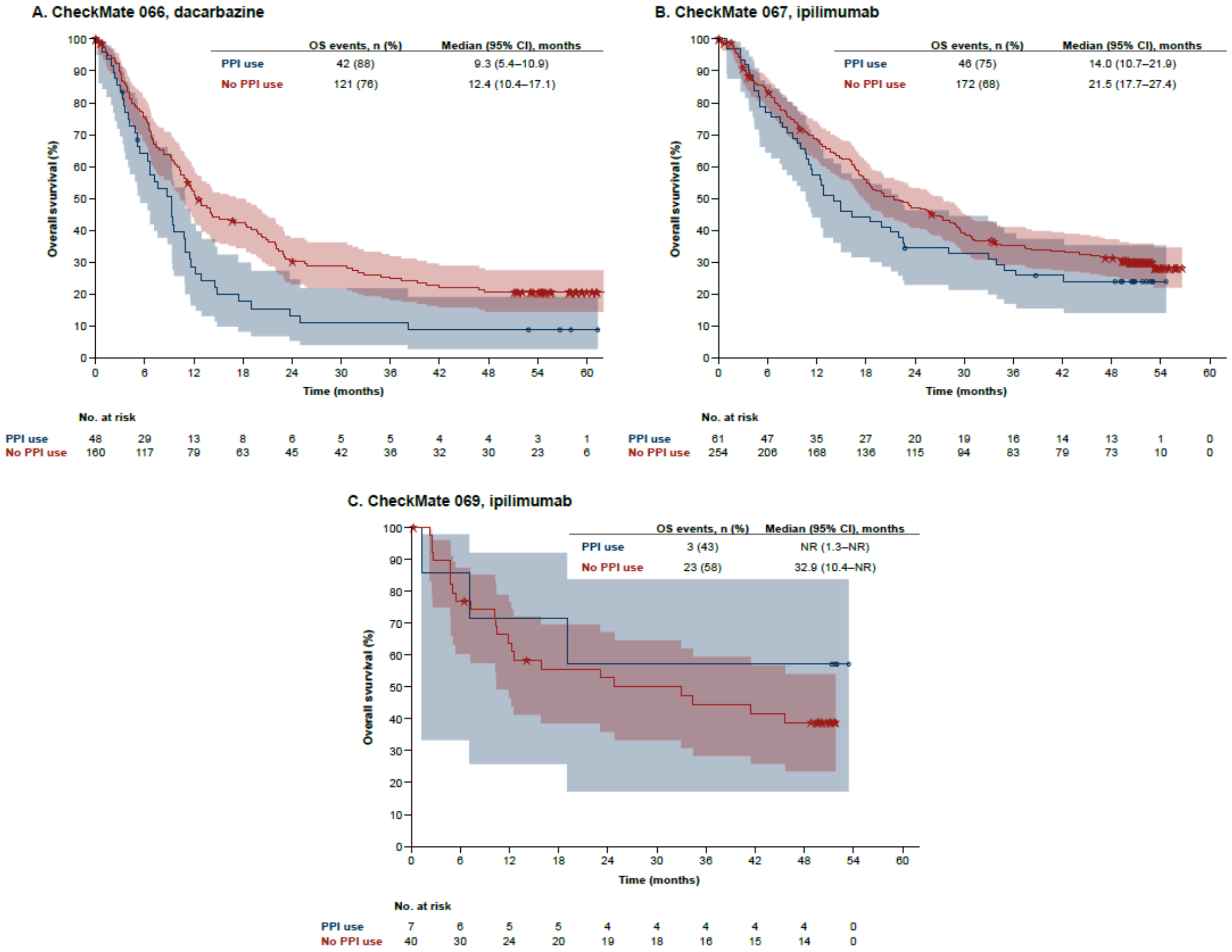

Figure S2. Overall survival by baseline PPI use in the control arms of CheckMate 066, 067, and 069. Kaplan–Meier estimates of overall survival in the dacarbazine arm of CheckMate 066 (**A**) and the ipilimumab arms of CheckMate 067 (**B**) and CheckMate 069 (**C**). Shaded areas are 95% log–log confidence bands. CI, confidence interval; OS, overall survival; PPI, proton pump inhibitor.
